# *Photobacterium arenosum* WH24, Isolated from the Gill of Pacific Oyster *Crassostrea gigas* from the North Sea of Germany: Co-cultivation and Prediction of Virulence

**DOI:** 10.1007/s00284-022-02909-2

**Published:** 2022-06-15

**Authors:** Hani Pira, Chandra Risdian, Mathias Müsken, Peter J. Schupp, Joachim Wink

**Affiliations:** 1 Microbial Strain Collection (MISG), Helmholtz Centre for Infection Research (HZI), 38124 Brunswick, Germany; 2 Research Unit for Clean Technology, National Research and Innovation Agency (BRIN), Bandung, 40135 Indonesia; 3 Central Facility for Microscopy, Helmholtz Centre for Infection Research (HZI), 38124 Brunswick, Germany; 4 Institute for Chemistry and Biology of the Marine Environment, University Oldenburg, Oldenburg, Germany

## Abstract

Cream colored bacteria from marine agar, strain WH24, WH77, and WH80 were isolated from the gill of the *Crassostrea gigas* a Pacific oyster with a filter-feeding habit that compels accompanying bacteria to demonstrate a high metabolic capacity, has proven able to colonize locations with changing circumstances. Based on the 16S rRNA gene sequence, all strains had high similarity to *Photobacterium arenosum* CAU 1568^T^ (99.72%). This study involved phenotypic traits, phylogenetic analysis, antimicrobial activity evaluation, genome mining, Co-cultivation experiments, and chemical studies of crude extracts using HPLC and LC-HRESIMS. *Photobacterium arenosum* WH24 and *Zooshikella harenae* WH53^T^were co-cultivated for 3 days in a rotary shaker at 160 rpm at 30 °C, and LC-MS monitored the chemical profiles of the co-cultures on the third day. The UV chromatograms of the extracts of the co-cultivation experiments show that *Zooshikella harenae* WH53^T^ could be inhibited by strain WH24. The high virulence of *Photobacterium arenosum* WH24 was confirmed by genome analysis. Gene groups with high virulence potential were detected: *tss*A (*Imp*A), *tss*B (*Imp*B/*vip*A), *tss*C (*Imp*C/*vip*B), *tss*E, *tss*F (*Imp*G/*vas*A), *tss*G (*Imp*H/vasB), *tss*M (*Icm*F/*vas*K), *tss*J (*vas*D), *tss*K (*Imp*J/*vas*E), *tss*L (*Imp*K/*vas*F), *clp*V (*tss*H), *vas*H, *hcp*, *lap*P, *plp*D, and *tps*B family.

**Supplementary Information:**

The online version contains supplementary material available at 10.1007/s00284-022-02909-2.

## Introduction

Strains of the genus *Photobacterium* are facultative aerobes and belong to the class of *gammaproteobacteria*. These Gram-negative bacteria can be present in various places, including fish guts, surfaces, light organs, free-living bacteria in the aquatic water column, and rotting animal tissue. Based on their genetic makeup, *Aliivibrio* and *Vibrio* are closely linked genera to *Photobacterium* [1]. The genus was first described by Beijerinck [2]. At the time of writing this article, the genus *Photobacterium* comprises 37 taxa with a validly published and correct name (https://www.bacterio.net/). Because of their luminous activity and use as a biosensor agent [3] and their ability to produce polyunsaturated fatty acids [4], antibacterial compounds [5], lipases [6], asparaginases [7], and esterases [8], *Photobacterium species* have been identified as an important group of bacteria. Humans can become sick by swallowing infected fish or bathing in brackish water when *Photobacterium* species enter person's urinary tract. Organ dysfunction, necrotizing fasciitis, and even death may occur in humans. Humans can tolerate the pathogen for up to 72 h. Antibiotics and radiation have both been used and attempted to cure the infection. Amputating the affected region of the body until the pathogen spreads is the safest option [9]. The majority of species are found in the light organs of various marine animals, including fish and squid. As pathogens or decomposers of deceased fish and commensals in the guts of many marine creatures, this form of connection might be characterized as symbiotic growth in the light organs of fish and squid [10]. However, it is known that luminous bacteria are widely distributed in marine habitats and can be found both free-living and host-associated. *Photobacterium*, a genus of light-producing marine bacteria, is an excellent illustration of this variety. It has been observed that certain isolates of *Photobacterium leiognathi* and *Photobacterium phosphoreum* have a particular genome to express the bioluminescent properties. These findings show that bioluminescence's regulation, nature, and ecological functions are likely to be diverse [11, 12]. Quorum sensing is frequently linked to bacterial bioluminescence control. According to Miller and Bassler, quorum sensing (abbreviated as QS) is the control of gene expression in response to changes in cell population density [13]. QS is a method of bacterial communication [14]. Actually, it's the bacteria's capacity to use tiny hormone-like chemical molecules called autoinducers to coordinate individual activity. QS was identified by examining bacterial bioluminescence [14]. QS has also been linked to virulence gene expression, biofilm development, swarming, antibiotic synthesis, and antibiotic resistance in bacteria. Our knowledge of the genetic, genomic, biochemical, and signal diversity of QS has advanced dramatically in recent years [14]. Bacteria employ a variety of techniques to interact with their surroundings and hosts. The majority of these interactions are dependent on protein synthesis and secretion. Protein secretion in bacteria is governed by various processes that are rather complicated and dependent on the organism's structure. As a result of changes in the structure of the bacterial cell walls and the bacterial cell membrane, this mechanism will be unique in positive and negative gram bacteria. Despite extensive study and significant progress in understanding secretion systems, the method and the structural and molecular mechanisms of these systems remain unknown. Specific secretion systems are a prerequisite for quorum sensing, so it is even more important to understand their individual components and how they work [15]. Type I to Type VI secretion systems (T1SS-T6SS) are known in Gram-negative bacteria. Different components, substances, and processes are found in each system. Materials must flow through both the inner and outer membranes of these bacteria, or specific compounds must enter the host cell, necessitating the use of a variety of molecules and processes [16]. Single-stage paths are Systems I, III, IV, and VI. These systems deliver the molecules they transport directly into the extracellular space without passing the periplasm. In two-stage secretion systems like II and V, proteins enter periplasm space with the aid of general secretion systems like Tat and Sec to find the appropriate folding and then find their way out via one of the two-stage secretion systems in the second phase [15].

One of the virulence factors in many pathogenic bacteria is The patatin-like protein D (*PlpD*) prototype of the subclass T5dSS, which secretes a lipolytic passenger that forms extracellular homodimers. This enzyme is released to a 16-stranded -barrel transporter, comparable to *TpsB* transporters seen in type Vb secretion systems [17].

Marine-derived bacteria have promise as a source of new bioactive chemicals, crucial for therapeutic development. However, like with terrestrial microbes, there is a high redundancy rate, resulting in the regular re-discovery of known chemicals. Under typical laboratory circumstances involving the development of axenic microbial strains, only a portion of the biosynthetic genes encoded by bacteria seems to be translated. Furthermore, most biosynthetic genes are not expressed in *vitro*, limiting the chemical variety of microbial chemicals created by fermentation. Co-cultivation (also known as mixed fermentation) of two or more distinct microbes, on the other hand, attempts to simulate the biological condition in which germs co-exist in complex microbial communities. During co-cultivation, the competition or antagonism results in greatly increased synthesis of constitutively present chemicals and/or a buildup of cryptic compounds not identified in the generating strain's axenic cultures [18]. Although this article does not address the pathogenicity of humans, we mentioned some important genomes with virulence effects. We isolated the *Photobacterium* species (WH24, WH77, and WH80) related *photobacterium arenosum* and analysed some elements that have an important role from ecological aspects, their secondary metabolite productions, co-cultivation, and important properties extracted from genome mining studies.

## Material and Methods

### Isolation

Oysters were taken from the Wilhelmshaven Sea in northern Germany in December 2019. (latitude: 53.5131; longitude: 08.14714). The strains WH24, WH77, and WH80 were isolated using the dilution plate method using the artificial seawater medium (ASW; ATI Coral Ocean) for 7 days at 30 °C from the gill of the Pacific oyster *Crassostrea gigas*. The ASW medium was supplemented with biotin (vitamin B7; 2 mg/L), nicotinic acid (20 mg/L), thiamine (vitamin B1; 10 mg/L), 4-aminobenzoic acid (10 mg/L), pantothenic acid (5 mg/L), pyridoxamine (vitamin B6; 50 mg/L), cyanocobalamin (vitamin B12; 20 mg/L), and cycloheximide (50 mg/mL) as antifungal agent. Individual cream-colored colonies were selected and transferred to Bacto marine agar (MA, Difco 2216), where they were purified by streaking on the same medium.

### Morphological, Physiological, and Biochemical Studies

Cells grown on MB (marine broth Difco 2216) media for 3 days at 30 °C were observed morphologically, including motility, using a light microscope (Zeiss Axio Sc pie. A1 microscope). Cells grown in MB media for 2 days at 30 °C were fixed with aldehydes (final concentrations: 5% formaldehyde and 2% glutaraldehyde), dehydrated in a gradient series of acetone, critical point dried, and coated with gold–palladium according to a previously published protocol [19]. At different magnifications, images were captured using a Zeiss Merlin field emission scanning electron microscope (FESEM) with a 25:75% ratio of Everhart–Thornley SE-detector and Inlens-SEM detector. On MA medium, growth was assessed over a variety of temperatures (4, 15, 20, 25, 30, 35, 40, and 45 °C) and pH values (pH 5, 6, 7, 8, 9, 10, and 11). Sodium chloride tolerance was determined using the following concentrations of NaCl (w/v): 0%, 2.5%, 5.0%, 7.5%, 10%, 15%, 25%, 30%; based on the method of Kutzner et al. [20]. A carbohydrate consumption study was conducted on ISP9 medium supplemented with 1% carbon sources [21] and 2.5% NaCl. All media were incubated for 7 days at 30 °C. Api Zym [22], Api Coryne [23], and Api 20E [24] stripes were used in the biochemical investigation. Antibiotic susceptibility testing was performed on MA medium for 48 h with the following antibiotics: polymyxin (50 µg/mL), gentamycin (50 µg/mL), oxytetracycline (10 µg/mL), ampicillin (100 µg/mL), chloramphenicol (30 µg/mL), spectinomycin (50 µg/mL), kanamycin (50 µg/mL), cephalosporin (50 µg/mL), fusidic acid (50 µg/mL), bacitracin (50 µg/mL), thiostrepton (50 µg/mL), trimethoprim (50 µg/mL), erythromycin (15 µg/mL), and tetracycline (50 µg/mL).

### 16S rRNA Gene Analysis

The Invisorb Spin Plant Mini Kit was used to extract genomic DNA following the instruction of the kit manufacturers (Stratec Molecular, Germany). PCR amplification of the 16S rRNA gene was performed according to Primahana et al. [25] with the primer F27 (5′AGAGTTTGATCMTGGCTCAG3′) and 1492R (5′TACGGYTACCTTGTTACGACTT-3′) [26]. The 16S rRNA gene was sequenced employing an Applied Biosystems 3730XL automated sequencer (ABI). BioEdit software was used to modify and assemble the sequence (version 7.0.5.3) [27]. The 16S rRNA gene sequence of strains WH24, WH77, and WH80 were almost completely sequenced (1,415 bp) and submitted in GenBank under the accession number MW888979, OM533648, and OM533649, respectively. The closest strains of strains WH24, WH77, and WH80 were identified based on 16S rRNA gene sequence similarity using the EZBioCloud system (https://www.ezbiocloud.net/) [28]. Based on Blast analysis (https://www.ncbi.nlm.nih.gov/) using 16S rRNA gene sequence, it was found that the 16S rRNA gene sequences of strain WH24 (1415 bp), WH77 (1343 bp), and WH80 (1262 bp) were 100% identical. Therefore, for further study, one of the strains (WH24) was used for evaluating its phenotypic and genotypic properties. Phylogenetic analysis of the 16S rRNA gene of strain WH24 with the closely related type strains was inferred by GGDC online server (http://ggdc.dsmz.de/) [29]. The sequence was analysed using a single-gene adaptation of the DSMZ phylogenomics program [30]. Multiple sequence alignment was performed using MUSCLE [31]. Randomised Axelerated Maximum Likelihood (RAxML) [32] and TNT (Tree analysis using New Technology) [33] programs were used to estimate Maximum likelihood (ML) and Maximum parsimony (MP) trees, respectively. For ML analysis, we used rapid bootstrapping with the autoMRE (extended majority rule) bootstrapping criteria [34]. In the case of MP, 1000 bootstrapping replicates were employed, and tree bisection and reconnection branch switching and ten random sequence addition repetitions. The X^2^ tests used in PAUP* (Phylogenetic Analysis using Parsimony*) were used to analyse the sequences [35].

### Chemotaxonomy

The biomass used in the chemical analyses was grown and collected for 7 days at 30 °C in a 250-mL flask containing 100 mL MB medium on a rotary shaker (160 revolutions per minute). The chemotaxonomic analysis was conducted on freeze-dried biomass. Minnikin’s technique [36] for obtaining isoprenoid quinones was adopted. The compounds were analysed using high-performance liquid chromatography fitted with diode-array detection and mass spectrometry (HPLC-DAD-MS) described by Risidian et al. [37], with some adjustments to the column, mobile phase, and flow rate. For isocratic conditions, solvent A (35% isopropanol + 1 percent water + 0.1% formic acid) and solvent B (65% acetonitrile + 1% water + 0.1% formic acid) were utilized at a flow rate of 0.3 mL/min. The isoprenoid quinones were separated using a Waters ACQUITY UPLC BEH C18 column (2.1 × 50 mm, 1.7 m). The extraction and methylation of fatty acids were carried out in accordance with Sasser's protocol [38]. Fatty acid methyl esters (FAME) were analysed using an Agilent 6890N gas chromatography fitted with a flame ionization detector (FID). The methyl esters of fatty acids were isolated using a Macherey Nagel Optima 5 column (5% phenyl, 95% dimethylpolysiloxane; 50 m length; 0.32 mm inner diameter; 0.25 m film thickness). Their retention periods were compared to standards (in-house reference standard) to identify specific fatty acid methyl esters.

### Whole-Genome Analysis

For whole-genome sequencing, Illumina's next-generation sequencing technology using MiSeq 600 cycle v3 was employed, and Unicycler was applied for genome de novo assembly [39]. The ContEst16S method was used to determine the purity of the 16S rRNA gene in the whole-genome data (https://www.ezbiocloud.net/tools/contest16s) [40]. The NCBI Prokaryotic Genome Annotation Pipeline (PGAP) was used to do automated genome annotation [41]. Additionally, the draft genome assembly was submitted for metabolic reconstruction analysis to the RAST (Rapid Annotation Using Subsystem Technology) database (https://rast.nmpdr.org/) [42]. Using the antiSMASH service (https://antismash.secondarymetabolites.org/), secondary metabolite gene clusters were predicted [43, 44]. Utilizing the Type (Strain) Genome Server (TYGS) (https://tygs.dsmz.de/https://tygs.dsmz.de/) [45], a phylogenomic tree was constructed using the whole-genome sequence of strain WH24 and its closest phylogenetic relatives. The Ezbiocloud and NCBI databases were used to extract whole-genome sequences of *Photobacterium arenosum* CAU 1568^T^, *Photobacterium galatheae* DSM 100496^T^, *Photobacterium ganghwense* strain JCM 12487^T^, Ph*otobacterium leiognathi* DSM 21260^T^, *Photobacterium phosphoreum* DSM 15556^T^, and *Photobacterium halotolerans* DSM 18316^T^. The strain WH24 genome sequence was submitted to the Type (Strain) Genome Server (TYGS) (https://tygs.dsmz.de; accessed on 05 February 2022). Genome BLAST Distance Phylogeny (GBDP) was used to make all pairwise comparisons for phylogenomic inference, and accurate intergenomic distances were determined using the 'trimming' procedure and distance formula d5 [29]. One hundred distance replicates were considered each. We utilized the Genome-to-Genome Distance Calculator (GGDC 2.1) to generate digital DDH (dDDH) values and confidence intervals using the recommended parameters (GGDC 2.1) [46]. From the resultant intergenomic distances with branch support, a balanced minimal evolution tree was inferred using FASTME 2.1.6.1, which included postprocessing for Subtree Pruning and Regrafting (SPR) [47]. The tree's branch support was calculated using 100 pseudobootstrap replications. The OrthoANIu algorithm [48] (https://www.ezbiocloud.net/tools/ani) was used to determine the average nucleotide identity (ANI) genome size and guanine and cytosine (G + C) content. The RAST algorithm v1.073 from the KBase database https://narrative.kbase.us/ genes (The genome features were functionally annotated using the following algorithms: Kmers V2; Kmers V1; protein similarity [49]) was applied to genome mining and annotated all following genes: the light-emission-involved genes-related Quorum sensing and bacterial luciferase and other important genes like *lux*R, *lux*I, *lux*A, *lux*B, *lux*C, *lux*D, *lux*E, *lux*F, *lux*G, *lux*O (Regulatory protein),*lux*S, *ssp*A, *mrc*A, *pnp*, *rib*D, *lum*P, *mia*A, *thy*A, *mdh*, *pls*X, *lum*Q, *suc*A, *aph*A, *cqs*A, *cqs*S, *lux*S, *rib*E [11, 12] and also the important genes involved in virulence of *Photobacterium* sp included Type I to Type VI secretion systems (T1SS-T6SS) like *Imp*A, t*ss*B (*Imp*B/*vip*A), *tss*C (*Imp*C/*vip*B), *tss*E, *tss*F (*Imp*G/*vas*A), *tss*G (*Imp*H/v*as*B), *tss*M (*Icm*F/v*as*K), *tss*J (*vas*D), *tss*K (*Imp*J/v*as*E), *tss*L (*Imp*K/*vas*F), *clp*V (*tss*H), *Imp*I/*Vas*C, *vas*H, *hcp*, *lap*P, *lap*L, secreted agglutinin (*rtx*), *plp*D, *tps*B family, *rst*A-*rst*B, *pdp*, *vsc*D, *vcr*D, *par*AB, *trac*, *frp*A, *tra*F [15, 50, 51].

The draft genome of strain WH24 was submitted to DDBJ/EMBL/GenBank with the accession number JAGSOZ000000000.

### Secondary Metabolite Production and Antimicrobial Activity

Growth of the strain WH24 was carried out for 5 days at 30 °C on a shaker in 250-ml Erlenmeyer flasks that contained 100 mL of MB medium with 2% (v/v) XAD-2 polymeric resin (160 revolutions per minute). Separation of XAD-2 was performed using a paper filter, and acetone was employed to extract secondary metabolites from the XAD-2. The extract was dried at 40 °C in a rotary evaporator. The dried extract was diluted in 1 mL methanol and evaluated for antimicrobial activity against a variety of bacteria: *Escherichia coli* wild type BW25113, *Escherichia coli* acrB JW25113, *Pseudomonas aeruginosa* DSM 19,882, *Staphylococcus aureus* Newman, *Citrobacter freundii* DSM 30,039, *Acinetobacter baumannii* DSM 30,008, *Bacillus subtilis* DSM 10, *Mycobacterium smegmatis* ATCC 700,084, *Mucor hiemalis* DSM 2656, *Wickerhamomyces anomalus* DSM 6766, and *Candida albicans* DSM 1665. The serial dilution procedure was used using 96-well microplates in accordance with Khosravi Babadi et al. [52].

### Co-culture Experiment

In this study, we co-cultivated the strains that were isolated in the same location in Oyster for instance, *Photobacterium* sp. strain WH24 and *Zooshikella harenae* WH53^T^ were cultivated [53] separately in 50 mL of MB Difco 2216 (Ref 279,110) liquid medium in a 125-mL Erlenmeyer flask. After 2 days of cultivation in a rotary shaker at 160 rpm at 30 °C, 2–5–8 mL volumes of the liquid cultures of WH24 and 2–5–5 mL of *Zooshikella harenae* WH53^T^ [53] were mixed and inoculated into a 250-mL baffled Erlenmeyer flask containing 100 mL of MB medium with 2% (v/v) XAD-2 polymeric resin medium, respectively. Mixed strains were Cocultivated for 3 days on a rotary shaker at 160 rpm at 30^◦^C, and LC-MS monitored the chemical profiles of the co-cultures on the third day.

### Base Peak Chromatogram Analysis of an Extract of Strain WH24

The analysis of the extract of strain WH24 was performed using an Agilent 1260 series HPLC-DAD system coupled with a MaXis ESI-TOF (Time of Flight) mass spectrometer (Bruker Daltonics, Bremen, Germany). The column C18 Acquity UPLC BEH (Ultra Performance Liquid Chromatography Ethylene Bridged Hybrid, Waters) was used as the stationary phase. The separation was carried out by gradient system employing two mobile phases (solvent A: H_2_O + 0.1% formic acid; solvent B: ACN + 0.1% formic acid) with the condition: 5% B (0.5 min), 5–100% B (0.5–20 min), and 100% B (20–25 min) and the flow rate was 0.6 mL/min (40 °C). Molecular formulas of the compounds were analysed using the Smart Formula algorithm (Bruker Daltonics) [54]. The selection of the major peaks of the base peak chromatogram (BPC) (cut-off intensity of 20%) was determined from the retention time of 1.5–18 min. The Dictionary of Natural Products database (DNP on USB, version 30.1, CRC Press, Taylor & Francis, Boca Raton, FL, USA) was used to predict the compounds based on the accurate mass with ± 0.01 Da.

## Results and Discussion

**Fig. 1:**
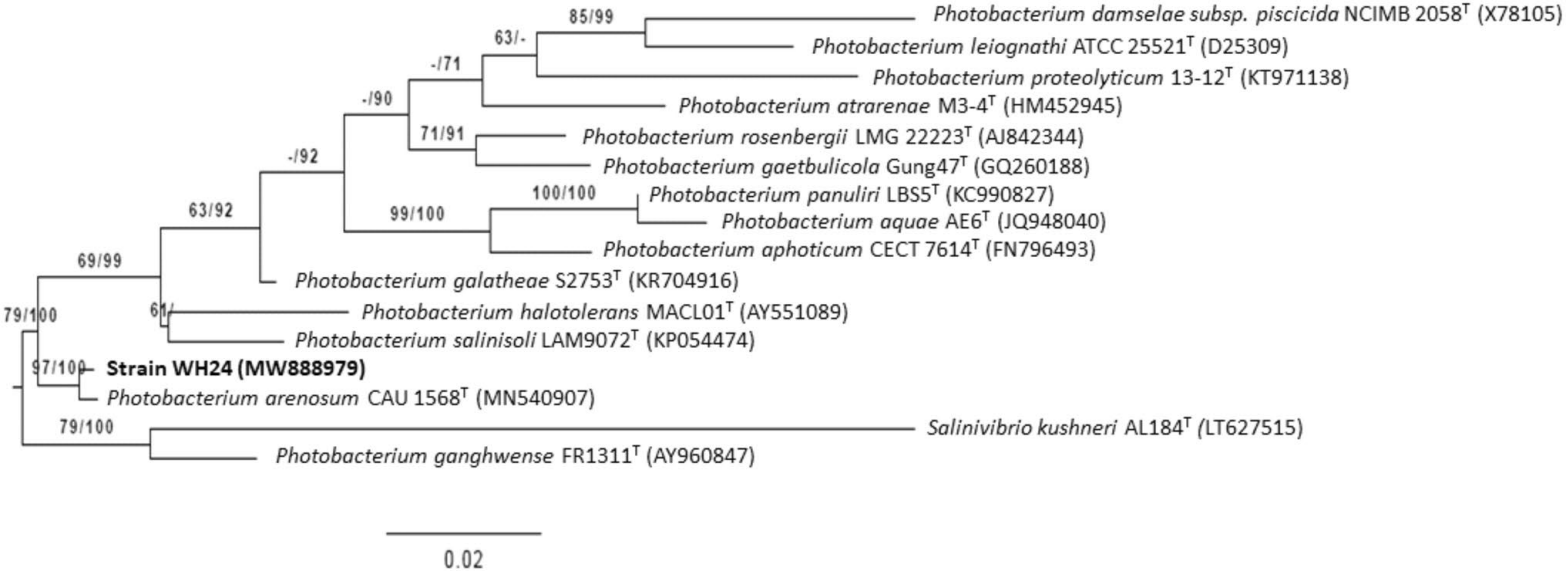
Phylogenetic tree based on 16S rRNA gene sequence of strain WH24 and type strains of the closely related species of the genus *Photobacterium*. The GTR + GAMMA model was used to infer the ML tree, which was then rooted using midpoint-rooting. The branches were scaled in terms of the expected number of substitutions per site. The numbers above the branches are support values when larger than 60% from ML (maximum likelihood, left) and MP (maximum parsimony, right) bootstrapping. The ML bootstrapping did not converge; hence 1000 replicates were conducted; the average support was 72.46%. MP analysis yielded the best score of 502 (consistency index 0.66, retention index 0.60) and 2 best trees. The MP bootstrapping average support was 86.38%

Microscopic observations show that strain WH24 is Gram-stain-negative and motile. The bacterium has single polar flagella and is rod-shaped with a cell size diameter of 0.5–0.8 µm in width and 1.5–2.6 µm in length (Supplementary Fig. S1). It seems to be an asporogenous bacterium. The optimal temperature for growth was determined to be 30 °C, and the optimal pH value was determined to be 7. On an agar medium without NaCl, low growth was identified. Tolerance to sodium chloride was up to 10%, with optimal growth occurring on conditions containing 2.5 and 5% sodium chloride. Biochemical properties based on Api ZYM, Api Coryne, and Api 20E assays that distinguish strain WH24 from its closest relatives are listed in Table S1, Table S2 and in the description of *Photobacterium arenosum*. All negative traits from commercial kits Api ZYM, Api Coryne, and Api 20E for *Photobacterium arenosum* WH24 are listed in Table S3. Strain WH24 could utilize mannitol, fructose, and cellulose as the sole carbon source. Strain WH24 was sensitive to polymyxin, gentamycin, chloramphenicol, thiostrepton, and erythromycin. However, the isolate was resistant to oxytetracycline, ampicillin, spectinomycin, kanamycin, cephalosporin, fusidic acid, bacitracin, trimethoprim, and tetracycline. According to Blast analysis, the 16S rRNA gene sequence of strain WH24 shows high similarity to *Photobacterium arenosum* CAU 1568^T^ (99.72%), *Photobacterium salinisoli* LAM9072^T^ (97.95%) and *Photobacterium halotolerans* MACL01^T^ (97.55%). In the phylogenetic tree, strain WH24 was located in the same clade with *P. arenosum* CAU 1568^T^ with a very high supported branch (Fig. 1). Based on whole-genome analysis, strain WH24 and *Photobacterium arenosum* CAU 1568^T^ had ANI values of 98.96% and dDDH scores of 90.8%, which are more than the species cut-off value of 95% and 70%, respectively [55]. Therefore, strain WH24 belongs to species *Photobacterium arenosum.* The major fatty acids identified in strain WH24 were C16:0 (21.79%), C16:1ω7c (17.11%), and C18:1ω7c (15.32%). The main polar lipids of strain WH24 were diphosphatidylglycerol (DPG), phosphatidylglycerol (PG), phosphatidylethanolamine (PE), unknown aminophospholipid (APL), unknown phospholipids (PL), and unknown polar lipid (L) (Supplementary Fig. S2). The major quinone of strain WH24 was ubiquinone-8 (Q-8).

**Fig. 2:**
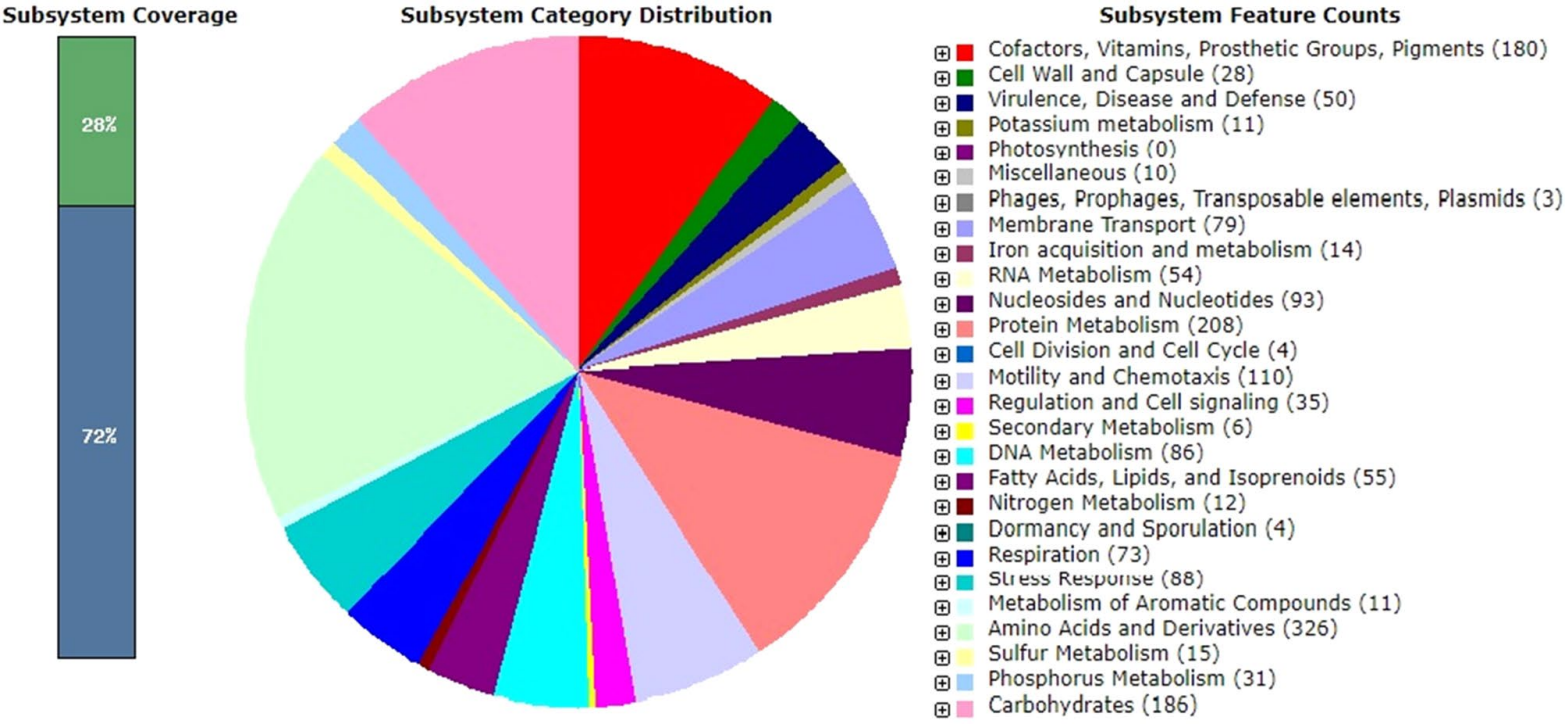
Subsystem category distribution of detected genes of strain *Photobacterium* sp. WH24 based on RAST annotation server (https://rast.nmpdr.org/)

The draft assembled genome sequence of strain WH24 consisted of 4,645,931 bp with a G + C content of 50.18% (GenBank accession No. JAGSOZ000000000). The genome included 4270 genes comprising 4181 protein-coding genes, 81 tRNA genes, 4 rRNA genes, and 4 non-coding RNA. According to the phylogenomic tree (Fig. S3), strain WH24 formed a clade with *Photobacterium arenosum* CAU 1568^T^. The results of genome mining by the Whole-genome showed just one difference for T6SS effector *tse* gene for strain WH24 isolated from Pacific Oyster from Germany and *Photobacterium arenosum* CAU 1568^T^. Other comparisons are determined and listed in Table 1. Based on RAST analysis, it was discovered that 28% of the genes were allocated to subsystems (Fig. 2). The largest number of predicted gene clusters concerned the metabolism of amino acids and derivatives (326), followed by protein metabolism (208) and carbohydrate metabolism (186). Genes responsible for motility and chemotaxis (110), metabolism of aromatic compounds (11), stress response (88), and dormancy and sporulation (4) were also detected. Eleven gene clusters involved in secondary metabolite production have been predicted by the antiSMASH server; five clusters were found to be more than 60% identical to known biosynthetic gene clusters: amonabactin P 750 biosynthetic gene cluster (85%), ectoine biosynthetic gene cluster (66%), rhizomide A, rhizomide B, rhizomide C biosynthetic gene cluster (100%), aerobactin biosynthetic gene cluster (88%), and pyrrolizixenamide A biosynthetic gene cluster (100%).

**Table 1:** Prediction of some important genes of *Photobacterium arenosum* WH24, *Photobacterium arenosum* CAU 1568.^T^, and closely related species using the KBase service

Genes	1	2	3	4	5	6	7
T6SS component *tss*A (*Imp*A)	+	** + **	** + **	** − **	** − **	** − **	** + **
T6SS component *tss*B (*Imp*B/*vip*A)	+	** + **	** + **	** − **	** − **	** − **	** + **
T6SS component *tss*C (*Imp*C/*vip*B)	+	+	+	** − **	** − **	** − **	+
T6SS lysozyme-like component *tss*E	+	+	+	** − **	** − **	** − **	+
T6SS component *tss*F (I*mp*G/*vas*A)	+	+	+	** − **	** − **	** − **	+
T6SS component *tss*G (*Imp*H/*vas*B)	+	+	+	** − **	** − **	** − **	+
T6SS component *tss*M (*Icm*F/*vas*K)	+	+	+	** − **	** − **	** − **	+
T6SS secretion lipoprotein *tss*J (*vas*D)	+	+	+	** − **	** − **	** − **	+
T6SS component *tss*K (*Imp*J/*vas*E)	+	+	+	** − **	** − **	** − **	+
T6SS outer membrane component *tss*L (*Imp*K/*vas*F)	+	+	+	** − **	** − **	** − **	+
T6SS AAA + chaperone *clp*V (*tss*H)	+	+	+	** − **	** − **	** − **	+
T6SS sigma-54-dependent regulator *vas*H	+	+	+	** − **	** − **	** − **	+
T6SS component *hcp*	+	+	+	** − **	** − **	** − **	+
T6SS effector *tse*	** − **	+	** − **	** − **	** − **	** − **	** − **
T6SS forkhead-associated domain protein *Imp*I/*Vas*C	** − **	** − **	+	** − **	** − **	** − **	** − **
T1SS-associated transglutaminase-like cysteine proteinase *lap*P, *lap*L	*lap*P	*lap*P	*lap*P	** − **	*lap*P *lap*L	*lap*L	** − **
T1SS secreted agglutinin (*rtx*)	** − **	** − **	+	** − **	+	+	** − **
T5SS Bifunctional outer membrane translocase / extracellular lipase, *plp*D	+	+	+	+	** − **	** − **	+
T5SS Channel-forming transporter/cytolysins activator of *tps*B family	+	+	** − **	** − **	** − **	** − **	+
T2SS *rst*A(phage-related replication protein)-*rst*B (phage-related integrase)	** − **	** − **	** − **	*rst*A	** − **	** − **	** − **
T3SS *pdp*, *vsc*D, *vcr*D, *par*AB, *trac*, *frp*A	** − **	** − **	** − **	** − **	** − **	** − **	** − **
T4SS *tra*F	** − **	** − **	** − **	** − **	** − **	** − **	** − **
Quorum-sensing regulator of virulence *hap*R	+	+	+	+	+	+	+
*lux*R family	+	+	+	+	+	+	+
*lux*I, *lux*A, *lux*B, *lux*C, *lux*D, *lux*E, *lux*F, *lux*G, *lux*O, *lux*S	*lux*O *lux*S	*lux*O *lux*S	*lux*O *lux*S	*lux*O *lux*S	*lux*A *lux*B *lux*C *lux*D *lux*E *lux*O *lux*S	*lux*A *lux*B *lux*C *lux*D *lux*E *lux*G *lux*O *lux*S	*lux*O *lux*S
*ssp*A, *mrc*A, *pnp*, *rib*D, *lum*P, *mia*A, *thy*A, *mdh*, *pls*X, *lum*Q, *suc*A, *aph*A, *cqs*A, *cqs*S, *rib*E	*pls*X *aph*A	*pls*X *aph*A	*pls*X *aph*A	*pls*X *aph*A	*pls*X *aph*A *cqs*A *cqs*S	*pls*X *cqs*A *cqs*S	*pls*X *aph*A

Strains: 1, Strain WH24; 2, *Photobacterium arenosum* CAU 1568^ T^; 3, *Photobacterium galatheae* DSM 100496^T^; 4, *Photobacterium ganghwense* strain JCM 12487^T^; 5, Ph*otobacterium leiognathi* DSM 21260^ T^; 6, *Photobacterium phosphoreum* DSM 15556^T^; 7, *Photobacterium halotolerans* DSM 18316^T^

− absent or not reported, + present or reported

**Fig. 3:**
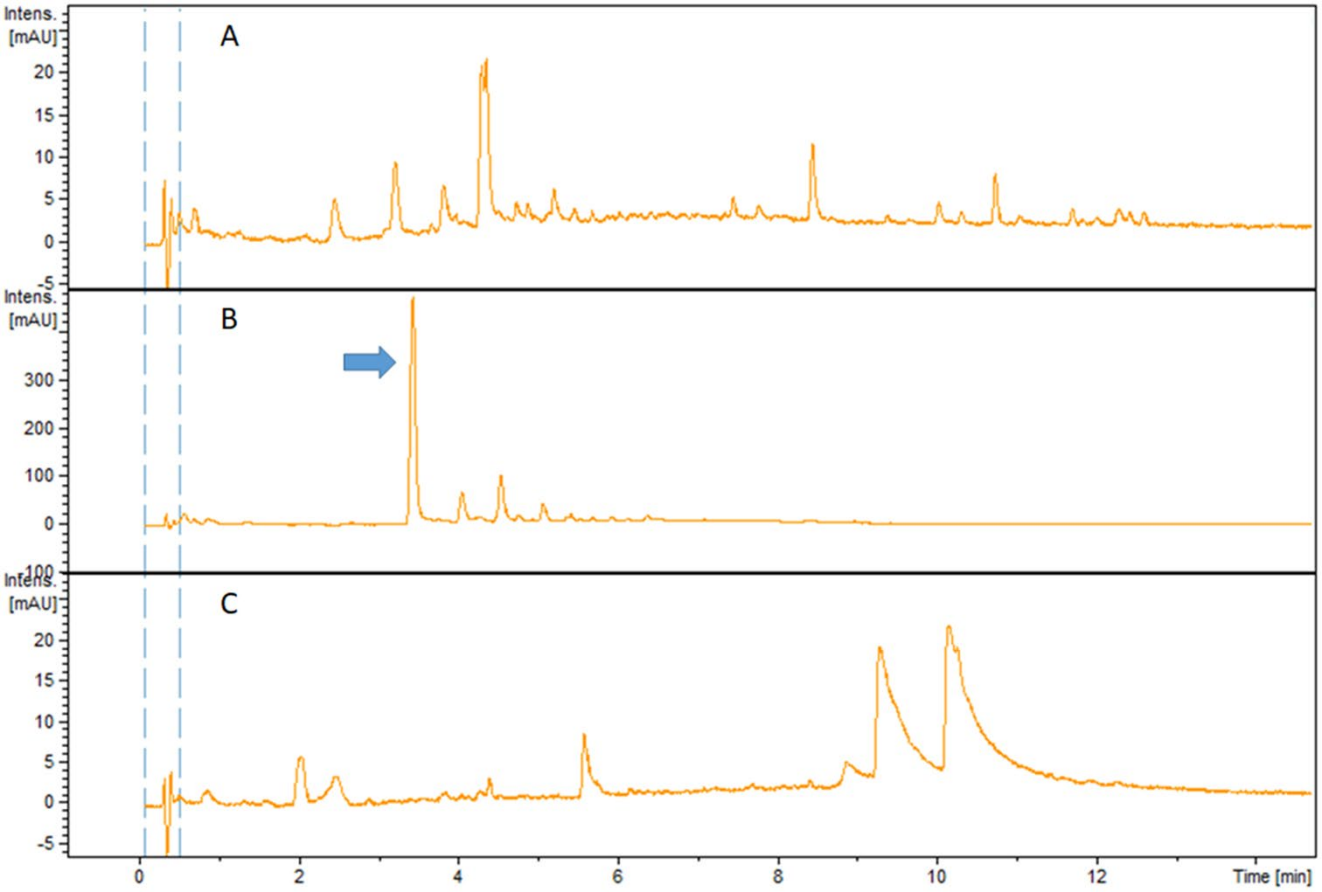
UV chromatogram of the extract of strain WH24 (**A**), UV chromatogram of the extract from co-cultivation of WH24 and *Z. harenae* WH53^T^ (**B**), and UV chromatogram of the extract from *Z. harenae* WH53^T^ (**C**). High peak produced by the co-cultivation was shown with an arrow (**B**). UV detection was conducted with the wavelength of 304 nm

**Fig. 4:**
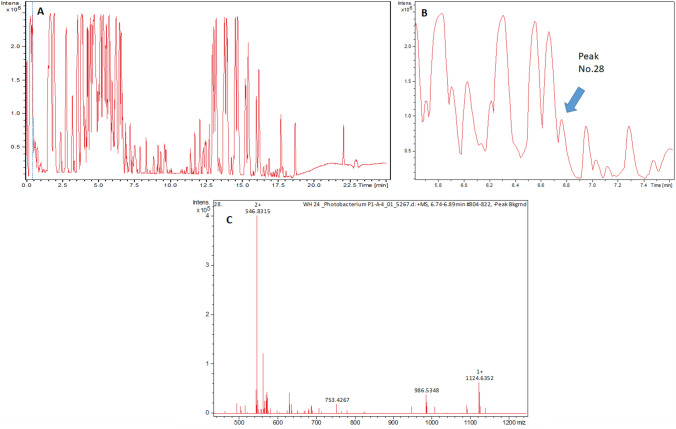
The base peak chromatogram (BPC) of the extract of strain WH24 (**A**), peak No. 28 which indicated a putatively new compound (**B**), and mass spectrum of peak No. 28 (**C**)

The extract of strain WH24 could strongly inhibit the growth of *Bacillus subtilis* DSM 10. Moderate growth inhibition was found against *Staphylococcus aureus* Newman, *Mycobacterium smegmatis* ATCC 700084; a weak growth inhibition was observed against *Candida albicans* DSM 1665, *Escherichia coli* acrB JW25113, *Mucor hiemalis* DSM 2656, *Wickerhamomyces anomalus* DSM 6766. There was no growth inhibition for *Acinetobacter baumannii* DSM 30008, *Citrobacter freundii* DSM30039, *Escherichia coli* BW 25113, and against *Pseudomonas aeruginosa* DSM 19882. From Table 2, it can be seen that the microbial activity of the extract derived from the co-cultivation mostly followed the bioactivity pattern of the extract from WH24. The UV chromatograms of the co-cultivation extracts also confirmed that peaks of *Zooshikella harenae* WH53^T^ [53] extracts could not be seen, suggesting that the growth of *Z. harenae* WH53^T^ [53] might be inhibited by strain WH24 (Fig. 3 and Table. S4). Interestingly, a high peak was detected in the extract from co-cultivation of the strains. The peak was identified at 3.43 min and possessed UV absorbance of 206 nm, 244 nm, 260 nm (sh), and 304 nm. The mass spectrum had major ion peaks of *m/z* 206.0805 [M + H]^+^ and *m/z* 228.0627 [M + Na]^+^. The calculated molecular size (M) was 205.0727, and in the Dictionary Natural Product database (DNP) 66 hits were found with the accurate mass of 205.0727 ± 0.01 Da. However, none of the hits was from *Photobacterium* nor *Zooshikella* source. There were 48 peaks detected as the major peaks in the BPC from the extract of strain WH24 (Fig. 4). The majority of the peaks had many hits from the Dictionary of Natural Products database (DNP), with the most hits being addressed to peak number 36 (12.98 min). However, many of them were not produced by *Photobacterium* species. There was only one peak (No. 28) that had no hit at all from the DNP, indicating that it could be a new compound. The peaks observed between 13.08 and 16.2 min were predicted as the compounds from kailuin groups. Kailuins are cyclic depsipeptides isolated from Gram-negative marine bacteria (*Vibrio* sp. and *Photobacterium halotolerans*) with cytotoxic properties. There are eight kailutin compounds with molecular weights ranging from around 697 to 754 [56–58]. The extract of strain WH24 contained the ions with *m/z* 698.4701 until *m/z* 754.5325; therefore, strain WH24 might produce all of the types of kailuins. The bioactivity of the extract of strain WH24 against *S. aureus* and *Mycobacterium smegmatis* still needs to be investigated, especially because of the presence of kailuins.

**Table 2:** Inhibition of test strains by extracts from cocultures of strain *Photobacterium arenosum* WH24 and *Zooshikella harenae* WH53.^T^ shown in MIC values (%)

Co-cultivated microorganisms	1	2	3	4	5	6	7	8	9	10	11
*Photobacterium arenosum* strain WH24	–	6.67	–	0.42	–	–	0.10	0.42	6.67	6.67	6.67
*Zooshikella harenae* WH53^T^	0.05	0.05	1.68	0.05	0.05	0.05	0.05	0.05	0.10	0.10	0.10
*P. arenosum* strain WH24 (5 mL) and *Z. harenae* WH53^T^ (5 mL)	–	–	–	0.42	–	–	–	1.68	3.37	0.42	–
*P. arenosum* strain WH24 (8 mL) and *Z. harenae* WH53^T^ (2 mL)	–	–	–	0.10	–	–	–	0.42	–	–	–
*P. arenosum* strain WH24 (2 mL) and *Z. harenae* WH53^T^ (5 mL)	–	1.68	–	0.10	–	–	–	0.42	–	–	–

Test strains: 1, *E. coli* wild type BW25113; 2, *E. coli* acrB JW25113; 3, *P. aeruginosa* DSM 19,882; 4, *S. aureus* Newman; 5, *C. freundii* DSM 30,039; 6, *A. baumannii* DSM 30,008; 7, *B.* subtilis DSM 10; 8, *M. smegmatis* ATCC 700,084; 9, *M. hiemalis* DSM 2656; 10, *W. anomalus* DSM 6766; 11, *C. albicans* DSM 1665

MIC value = 6.67–3.34% (low activity); 1.67–0.42% (moderate activity); and 0.21–0.05% (strong activity)

## Conclusion

According to the genome analysis of *Photobacterium arenosum* resulting in the detection of the following genes: T6SS component *tss*A (*Imp*A), T6SS component *tss*B (*Imp*B/*vip*A), T6SS component *tss*C (*Imp*C/*vip*B), T6SS lysozyme-like component *tss*E, T6SS component *tss*F (*Imp*G/*vas*A), T6SS component *tss*G (*Imp*H/*vas*B), T6SS component *tss*M (*Icm*F/*vas*K), T6SS secretion lipoprotein *tss*J (*vas*D), T6SS component *tss*K (*Imp*J/*vas*E), T6SS outer membrane component *tss*L (*Imp*K/*vas*F), T6SS AAA + chaperone *clp*V (*tss*H), T6SS sigma-54-dependent regulator *vas*H, T6SS component *hcp,* T1SS-associated transglutaminase-like cysteine proteinase *lap*P, T5SS Bifunctional outer membrane translocase / extracellular lipase, *plp*D, T5SS Channel-forming transporter/cytolysins activator of *tps*B family, we estimated that *Photobacterium arenosum* CAU 1568^T^ and WH24 both have high virulence potential. we reported no genes involving virulence factors in Ph*otobacterium leiognathi* DSM 21260^T^ and *Photobacterium phosphoreum* DSM 15556^T^, which are clear examples of bioluminescence in this genus. Whoever, it can be concluded that this type of bacterium can be a potential cause of disease in marine animals, as possible as the presence of Quorum-sensing regulator of virulence *hap*R, *lux*R family, and other genes might control gene expression in response to changes in cell population density. On the other hand, analysis of the Co-culture experiment for this study showed that these bacteria would produce new secondary metabolites and inhibit the target bacteria if they grow together. The UV chromatograms of the co-cultivation extracts also confirmed that *Zooshikella harenae* WH53^T^ could be inhibited by strain WH24. Therefore, further study on these bacteria as well as their interaction with marine organisms in the aquatic environment is recommended.

## Supplementary Information

Below is the link to the electronic supplementary material.

Supplementary file1 (DOCX 603 kb)

## Data Availability

Not applicable.
