# Supplementary material for: Photobacterium arenosum WH24, Isolated from the Gill of Pacific Oyster Crassostrea gigas from the North Sea of Germany: Co-cultivation and Prediction of Virulence
Source: Curr Microbiol. 2022 Jun 15;79(8):219. doi: 10.1007/s00284-022-02909-2 (PMC9200695; doi:10.1007/s00284-022-02909-2)
Supplement: Supplementary file 1 — Supplementary file1 (DOCX 603 kb) [file 284_2022_2909_MOESM1_ESM.docx]

***Photobacterium arenosum* WH24, isolated from gill of Pacific oyster *Crassostrea gigas* from the North Sea of Germany Cocultivation and prediction of virulency**

**Hani Pira**

Microbial Strain Collection (MISG), Helmholtz Centre for Infection Research (HZI), 38124 Braunschweig, Germany. ORCID iD: <https://orcid.org/0000-0001-9560-1287>

**Chandra Risdian**

Microbial Strain Collection (MISG), Helmholtz Centre for Infection Research (HZI), 38124 Braunschweig, Germany.

Research Unit for Clean Technology, National Research and Innovation Agency (BRIN), Bandung 40135, Indonesia

ORCID iD: <https://orcid.org/0000-0001-8997-7851>

**Mathias Müsken**

Central Facility for Microscopy, Helmholtz Centre for Infection Research (HZI), 38124 Braunschweig, Germany

ORCID iD: <https://orcid.org/0000-0002-5624-1378>

**Peter J. Schupp**

University Oldenburg, Institute for Chemistry and Biology of the Marine Environment, Oldenburg, Germany

ORCID iD: <https://orcid.org/0000-0003-4831-2751>

**Joachim Wink**

Microbial Strain Collection (MISG), Helmholtz Centre for Infection Research (HZI), 38124 Braunschweig, Germany. Phone number: +49 531 6181-4223 Email: [Joachim.Wink@helmholtz-hzi.de](mailto:Joachim.Wink@helmholtz-hzi.de)

ORCID iD: <https://orcid.org/0000-0001-9675-0276>

**Submitted to Current microbiology**


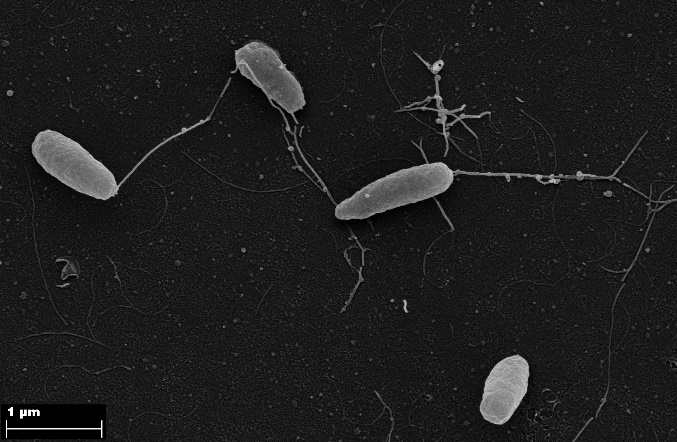


**Fig. S1:** Scanning electron microscopy images of strain WH24 after 3 days


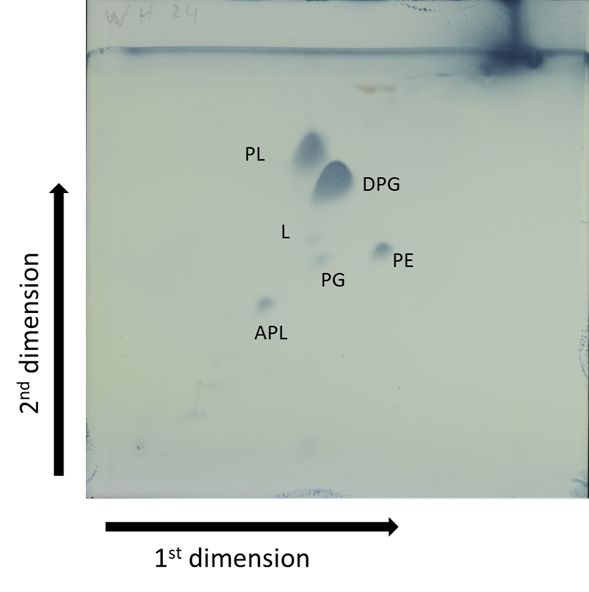


**Fig. S2:** Polar lipid detected in strain WH24. DPG: diphosphatidylglycerol; PG: phosphatidylglycerol; PE: phosphatidylethanolamine; APL: unknown aminophospholipid; PL: unknown phospholipid; and L: unknown polar lipid.


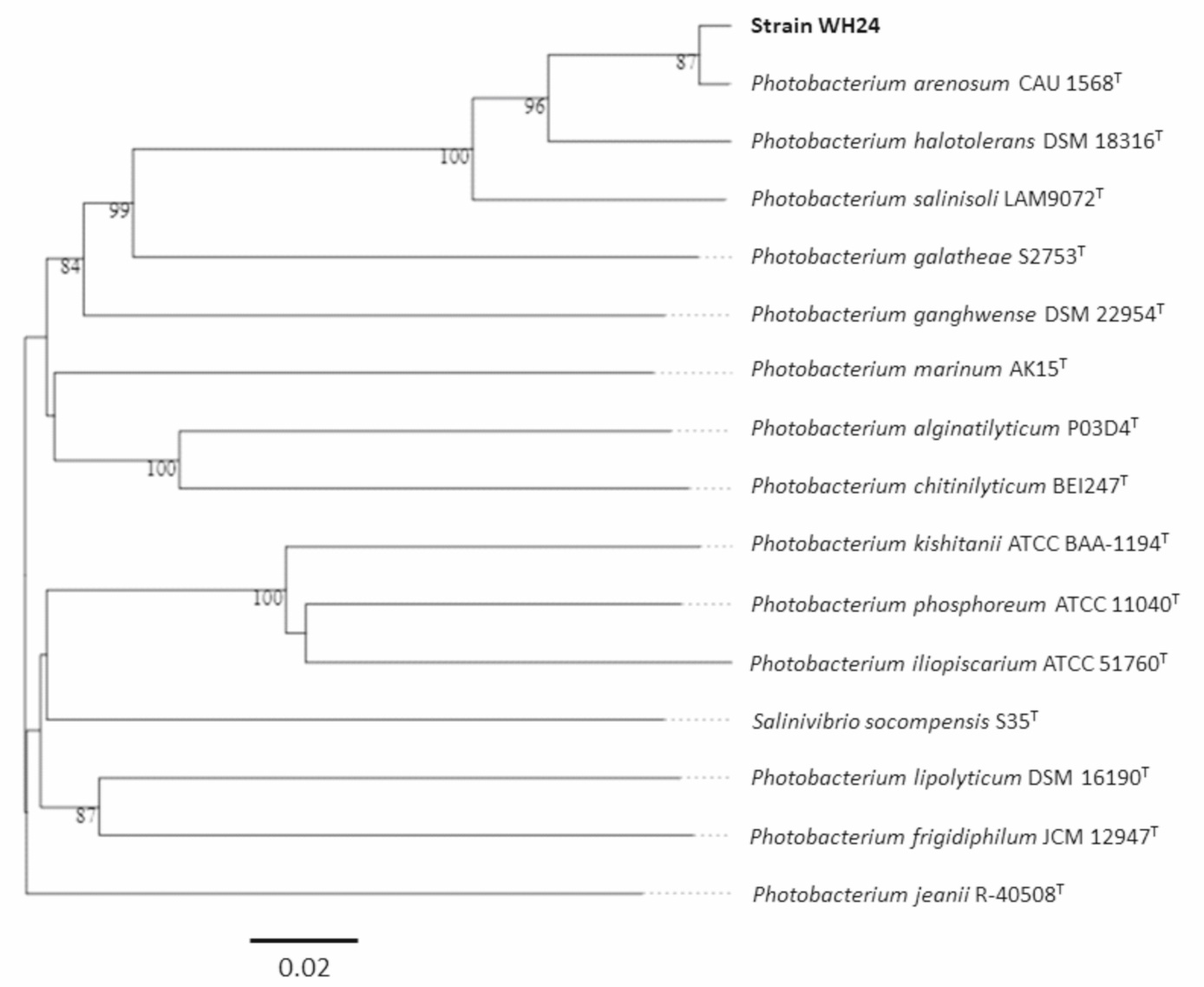


**Fig. S3:** Phylogenomic tree showing relationships between strain WH24 and the most closely related type strains from the genus *Photobacterium*. The number at the nodes are GBDP pseudo-bootstrap support values > 60 % from 100 replications, with average branch support of 73.8 %. Bar, 0.02 substitutions per site.

**Table S1**: The presence or absence of various enzymes in strain WH24 as detected by the Api ZYM system

| **Enzyme** | **Observation** | **Enzyme** | **Observation** |
| --- | --- | --- | --- |
| Phosphatase alkaline | (+) | Naphtol-AS-BI-phosphohydrolase | - |
| Esterase (C4) | (+) | α-galactosidase | - |
| Esterase lipase (C8) | + | β-galactosidase | - |
| Lipase (C14) | (+) | β-glucurunidase | - |
| Leucine arylamidase | ++ | α-glucosidase | - |
| Valine arylamidase | (+) | β-glucosidase | - |
| Cystine arylamidase | - | N-acetyl-beta- glucosaminidase | ++ |
| Trypsin | - | α-mannosidase | - |
| Chymotrypsin | - | α-fucosidase | - |
| Phosphatase acid | - |  |  |
| ++ strong; + good; (+) weak; - no activity | | | |

**Table S2:** Phenotypic characteristics that distinguish strain WH24 from the most closely related *Photobacterium* species. Strains: 1, WH24; 2, *Photobacterium arenosum* CAU 1568^T^ ; 3, *Photobacterium halotolerans* DSM 18316^T^; 4, *Photobacterium galatheae* DSM 100496^T^; 5, *Photobacterium salinisoli* LAM9072^T^.

| **Characteristics** | **1** | **2** | **3** | **4** | **5** |
| --- | --- | --- | --- | --- | --- |
| Temperature range for growth (°C) | 8-38 | 4-37** | 4-37* | 15-40† | 10-40‡ |
| pH spectrum for growth | 5.0-10.0 | 6.0-10.5** | 5.0-8.5* | 6.0-10.0† | 6.0-11.0‡ |
| NaCl range for growth (%) | 2.5-10.0 | 0.0-6.0** | 0-10.0 | 0-7.5 | 0-0.9‡ |
| Cystine arylamidase | - | -** | - | - | (+)‡ |
| α-Glucosidase | - | -** | - | - | +‡ |
| Alkaline phosphatase | (+) | +** | + | ++ | +‡ |
| Esterase (C4) | (+) | -** | (+) | (+) | +‡ |
| Lipase (C14) | (+) | -** | + | (+) | (+)‡ |
| Valine arylamidase | (+) | +** | ++ | (+) | +‡ |
| Phosphatase acid | - | -** | ++ | ++ | +‡ |
| Naphtol-AS-BI-phosphohydrolase | - | +** | (+) | (+) | (+)‡ |
| Reduction of nitrate to nitrite | - | -** | + | + | -‡ |
| Urease | + | -** | + | + | -‡ |
| Gelatin(hydrolysis) | + | -** | + | + | -‡ |
| Ribose fermentation | - | ND | + | + | ND |
| Xylose fermentation | - | -** | + | - | +‡ |
| Mannitol fermentation | + | ND | + | - | +‡ |
| Maltose fermentation | - | -** | + | + | +‡ |
| Sucrose fermentation | - | -** | + | - | +‡ |
| Glycogen fermentation | - | ND | + | + | +‡ |
| Arginine dihydrolase | + | -** | + | + | -‡ |
| Polar lipids | DPG, PG, PE, APL, PL, L | DPG, PG, PE, PL, APL,L,PC** | DPG, PG, PE, AL, APL, PL, L | DPG, PG, PE, AL, APL, PL | DPG‡, PG ‡, PE ‡, APL‡, L‡ |
| Major fatty acid | C_16:0_  C_16:1_ω7c C_18:1_ω7c | C_16:0_  ** C_16:1_ω7c** C_18:1_ω7c** | C_16:0_ C_16:1_ ω7c C_18:1_ ω7c | C_16:0_ C_16:1_ ω7c C_18:1_ ω7c | C_16 : 0_‡ C_16 : 1_ ω 6c‡ C_16 : 1_ ω7c‡ C_18 : 1_ ω7c‡ C_18 : 1_ ω 6c‡ |
| Contigs | 68 | 38** | 62 | 80 | 26 |
| No. of protein | 4,159 | 4,233** | 4,012 | 3,967 | 4,379 |
| rRNA | 4 | 3** | 3 | 3 | 20 |
| tRNA | 81 | 92** | 89 | 99 | 96 |
| No. of Gene | 4,270 | 4,257** | 4,185 | 4,111 | 4,533 |
| Other RNA | 4 | ND | 5 | 6 | 4 |
| Pseudogene | 22 | ND | 76 | 36 | 34 |
| G+C content (mol%) | 50.18 | 50.10** | 50.91 | 49.55 | 50.16 |
| Total sequence length (Mbp ) | 4.64 | 4.8** | 4.68 | 4.53 | 4.73 |

++ strong; + good; (+) weak; - no activity ; ND no data

*Data from Rivas et al. [59] **Data from Weerawongwiwat et al. [[6](#Weerawongwiwat60)0] s †Data from Machado et al. [[61](#Machado61)] ‡Data from Li et al. [[62](#Li62)] No data (ND), Diphosphatidylglycerol (DPG), phosphatidylglycerol (PG), phosphatidylethanolamine (PE), unidentified aminolipid (AL), unidentified aminophospholipid (APL), phosphatidylcholine(PC), unidentified phospholipids (PL), and unidentified polar lipid (L)

**Table S3:** List all negative traits from commercial kits Api ZYM, Api Coryne, and Api 20E for *Photobacterium arenosum* WH24

| Strains | Activity |
| --- | --- |
| *Photobacterium arenosum* WH24 |  |
| cystine arylamidase, trypsin, α-chymotrypsin, α-galactosidase, β-galactosidase, β-glucuronidase, α-glucosidase, α-mannosidase, α-fucosidase, acid phosphatase, pyrazinamidase, pyrrolidonyl arylamidase, esculin (β-glucosidase), lactose fermentation, Naphtol-AS-BI-phosphohydrolase, ribose fermentation, xylose fermentation, maltose fermentation, sucrose fermentation, glycogen fermentation, Onitrophenyl-ß-D-galactopyranoside, production of hydrogen sulfide, tryptophan deaminase, indole, detection of acetoin (acetyl methylcarbinol), fermentation of inositol, fermentation of sorbitol, fermentation of rhamnose, fermentation of melibiose, fermentation of amygdalin, fermentation of mannose, Reduction of nitrate to nitrite | - |

- no activity

**Table S4:** Peak analysis of the base peak chromatogram (BPC) of the extract of strain WH24

| **Compound** | **RT (min)** | **Major Ion** | | **DNP Result (hits)** | **DNP Result (hits) with Photobacterium as the biological sources** |
| --- | --- | --- | --- | --- | --- |
|  |  | **m/z** | **Formula** |  |  |
| 1 | 1.78 | 261.1233 | [M+H]^+^ | 21 | 0 |
|  |  | 283.1052 | [M+Na]^+^ |  |  |
| 2 | 2.03 | 197.1282 | [M+H]^+^ | 21 | 0 |
|  |  | 219.1102 | [M+Na]^+^ |  |  |
| 3 | 2.48 | 229.1002 | [M+H]^+^ | 85 | 0 |
|  |  | 251.0824 | [M+Na]^+^ |  |  |
| 4 | 2.86 | 229.1003 | [M+H]^+^ | 85 | 0 |
|  |  | 251.0824 | [M+Na]^+^ |  |  |
| 5 | 3.29 | 603.3137 | [M+H]^+^ | 9 | 0 |
|  |  | 625.2956 | [M+Na]^+^ |  |  |
|  |  | 302.1603 | [M+2H]^2+^ |  |  |
| 6 | 3.66 | 674.3522 | [M+H]^+^ | 7 | 0 |
|  |  | 696.3328 | [M+Na]^+^ |  |  |
| 7 | 3.94 | 397.2079 | [M+H]^+^ | 99 | 0 |
|  |  | 419.1899 | [M+Na]^+^ |  |  |
| 8 | 4 | 553.3342 | [M+H]^+^ | 21 | 0 |
|  |  | 575.3162 | [M+Na]^+^ |  |  |
| 9 | 4.08 | 698.4082 | [M+H]^+^ | 3 | 0 |
|  |  | 720.3900 | [M+Na]^+^ |  |  |
| 10 | 4.19 | 546.2922 | [M+H]^+^ | 4 | 0 |
|  |  | 568.2742 | [M+Na]^+^ |  |  |
| 11 | 4.31 | 425.2758 | [M+H]^+^ | 38 | 0 |
|  |  | 447.2577 | [M+Na]^+^ |  |  |
| 12 | 4.39 | 484.3129 | [M+H]^+^ | 5 | 0 |
|  |  | 506.2949 | [M+Na]^+^ |  |  |
| 13 | 4.54 | 652.4029 | [M+H]^+^ | 2 | 0 |
|  |  | 674.3844 | [M+Na]^+^ |  |  |
| 14 | 4.65 | 491.2865 | [M+H]^+^ | 9 | 0 |
|  |  | 513.2684 | [M+Na]^+^ |  |  |
| 15 | 4.79 | 576.3504 | [M+H]^+^ | 4 | 0 |
|  |  | 598.3322 | [M+Na]^+^ |  |  |
| 16 | 5.05 | 755.3933 | [M+H]^+^ | 13 | 0 |
|  |  | 777.3744 | [M+Na]^+^ |  |  |
|  |  | 378.2001 | [M+2H]^2+^ |  |  |
| 17 | 5.12 | 553.3343 | [M+H]^+^ | 21 | 0 |
|  |  | 575.3166 | [M+Na]^+^ |  |  |
| 18 | 5.26 | 439.2915 | [M+H]^+^ | 61 | 0 |
|  |  | 461.2768 | [M+Na]^+^ |  |  |
| 19 | 5.43 | 965.5666 | [M+H]^+^ | 2 | 0 |
|  |  | 987.5484 | [M+Na]^+^ |  |  |
|  |  | 483.2866 | [M+2H]^2+^ |  |  |
| 20 | 5.63 | 838.4458 | [M+H]^+^ | 5 | 0 |
|  |  | 860.4277 | [M+Na]^+^ |  |  |
|  |  | 419.7262 | [M+2H]^2+^ |  |  |
| 21 | 5.71 | 939.5410 | [M+H]^+^ | 13 | 0 |
|  |  | 470.2739 | [M+2H]^2+^ |  |  |
| 22 | 5.83 | 530.2975 | [M+H]^+^ | 3 | 0 |
|  |  | 552.2796 | [M+Na]^+^ |  |  |
| 23 | 5.91 | 1001.509 | [M+H]^+^ | 4 | 0 |
|  |  | 1023.4907 | [M+Na]^+^ |  |  |
|  |  | 501.2578 | [M+2H]^2+^ |  |  |
| 24 | 6.03 | 657.3968 | [M+H]^+^ | 3 | 0 |
|  |  | 679.3789 | [M+Na]^+^ |  |  |
| 25 | 6.31 | 790.4137 | [M+H]^+^ | 3 | 0 |
|  |  | 395.71 | [M+2H]^2+^ |  |  |
|  |  | 1100.5776 | [M+H]^+^ | 2 | 0 |
|  |  | 550.792 | [M+2H]^2+^ |  |  |
| 26 | 6.55 | 552.3756 | [M+H]^+^ | 2 | 0 |
|  |  | 574.3577 | [M+Na]^+^ |  |  |
| 27 | 6.67 | 889.4818 | [M+H]^+^ | 26 | 0 |
|  |  | 911.4636 | [M+Na]^+^ |  |  |
|  |  | 445.2441 | [M+2H]^2+^ |  |  |
| 28 | 6.76 | 1092.6564 | [M+H]^+^ | 0 | 0 |
|  |  | 546.8315 | [M+2H]^2+^ |  |  |
|  |  | 1124.6352 | [M+H]^+^ | 0 | 0 |
|  |  | 562.8209 | [M+2H]^2+^ |  |  |
| 29 | 6.95 | 538.3598 | [M+H]^+^ | 3 | 0 |
|  |  | 560.3412 | [M+Na]^+^ |  |  |
| 30 | 7.29 | 241.1908 | [M+H]^+^ | 0 | 0 |
|  |  | 263.1729 | [M+Na]^+^ |  |  |
| 31 | 8.38 | 255.2066 | [M+H]^+^ | 3 | 0 |
|  |  | 277.1886 | [M+Na]^+^ |  |  |
| 32 | 11.76 | 670.4385 | [M+H]^+^ | 4 | 0 |
|  |  | 692.4205 | [M+Na]^+^ |  |  |
| 33 | 12.11 | 730.4418 | [M+H]^+^ | 1 | 0 |
|  |  | 752.4237 | [M+Na]^+^ |  |  |
| 34 | 12.49 | 684.4544 | [M+H]^+^ | 1 | 0 |
|  |  | 706.4363 | [M+Na]^+^ |  |  |
| 35 | 12.76 | 744.4576 | [M+H]^+^ | 1 | 0 |
|  |  | 766.4395 | [M+Na]^+^ |  |  |
| 36 | 12.98 | 279.1589 | [M+H]^+^ | 140 | 0 |
|  |  | 301.1409 | [M+Na]^+^ |  |  |
| 37 | 13.08 | 698.4701 | [M+H]^+^ | 2 | 1 (kailuin A) |
|  |  | 720.4518 | [M+Na]^+^ |  |  |
| 38 | 13.23 | 698.4703 | [M+H]^+^ | 2 | 1 (kailuin A) |
|  |  | 720.4518 | [M+Na]^+^ |  |  |
| 39 | 13.81 | 712.4858 | [M+H]^+^ | 1 | 0 |
|  |  | 734.4674 | [M+Na]^+^ |  |  |
| 40 | 13.97 | 712.486 | [M+H]^+^ | 1 | 0 |
|  |  | 734.4675 | [M+Na]^+^ |  |  |
|  |  | 724.4857 | [M+H]^+^ | 1 | 1 (kailuin F) |
|  |  | 746.4674 | [M+Na]^+^ |  |  |
| 41 | 14.57 | 726.5014 | [M+H]^+^ | 2 | 2 (kailuin B; Kailuin C) |
|  |  | 748.483 | [M+Na]^+^ |  |  |
| 42 | 14.73 | 726.5015 | [M+H]^+^ | 2 | 2 (kailuin B; Kailuin C) |
|  |  | 748.4829 | [M+Na]^+^ |  |  |
| 43 | 15.25 | 740.5169 | [M+H]^+^ | 2 | 1 (kailuin G) |
|  |  | 762.4987 | [M+Na]^+^ |  |  |
|  |  | 752.5166 | [M+H]^+^ | 1 | 1 (kailuin D) |
|  |  | 774.4983 | [M+Na]^+^ |  |  |
| 44 | 15.29 | 740.5169 | [M+H]^+^ | 2 | 1 (kailuin G) |
|  |  | 762.4988 | [M+Na]^+^ |  |  |
| 45 | 15.47 | 740.5169 | [M+H]^+^ | 2 | 1 (kailuin G) |
|  |  | 762.4988 | [M+Na]^+^ |  |  |
| 46 | 16.03 | 754.5325 | [M+H]^+^ | 2 | 2 (kailuin E; kailuin H) |
|  |  | 776.5144 | [M+Na]^+^ |  |  |
| 47 | 16.2 | 754.5324 | [M+H]^+^ | 2 | 2 (kailuin E; kailuin H) |
|  |  | 776.5143 | [M+Na]^+^ |  |  |
| 48 | 17.72 | 363.2506 | [M+Na]^+^ | 122 | 0 |
|  |  | 703.5122 | [2M+Na]^+^ |  |  |
